# Rapid and Efficient Clearance of Blood-borne Virus by Liver Sinusoidal Endothelium

**DOI:** 10.1371/journal.ppat.1002281

**Published:** 2011-09-29

**Authors:** Latha P. Ganesan, Sudhasri Mohanty, Jonghan Kim, K. Reed Clark, John M. Robinson, Clark L. Anderson

**Affiliations:** 1 Department of Internal Medicine, The Ohio State University, Columbus, Ohio, United States of America; 2 Harvard School of Public Health, Boston, Massachusetts, United States of America; 3 Department of Pediatrics, The Ohio State University, Columbus, Ohio, United States of America; 4 Center for Gene Therapy, Research Institute at Nationwide Children's Hospital, Columbus, Ohio, United States of America; 5 Department of Physiology and Cell Biology, The Ohio State University, Columbus, Ohio, United States of America; Harvard University, United States of America

## Abstract

The liver removes quickly the great bulk of virus circulating in blood, leaving only a small fraction to infect the host, in a manner characteristic of each virus. The scavenger cells of the liver sinusoids are implicated, but the mechanism is entirely unknown. Here we show, borrowing a mouse model of adenovirus clearance, that nearly all infused adenovirus is cleared by the liver sinusoidal endothelial cell (LSEC). Using refined immunofluorescence microscopy techniques for distinguishing macrophages and endothelial cells in fixed liver, and identifying virus by two distinct physicochemical methods, we localized adenovirus 1 minute after infusion mainly to the LSEC (∼90%), finding ∼10% with Kupffer cells (KC) and none with hepatocytes. Electron microscopy confirmed our results. In contrast with much prior work claiming the main scavenger to be the KC, our results locate the clearance mechanism to the LSEC and identify this cell as a key site of antiviral activity.

## Author Summary

The liver has long been known as the garbage dump of the body, capable of rapidly removing hazardous pathogens and useless particles from the blood stream, thereby protecting the host. The only cell doing the removal has been thought to be the liver's macrophages. This is likely true for larger particles such as bacteria. But for smaller particles the size of virus or small antibody-antigen complexes, macrophages are probably not the cell responsible for the bulk of removal. We suggest, rather, it is the endothelial cell of the liver's blood circulatory system that takes up and destroys the majority of virus, doing so quickly (minutes) and extensively (>90%), leaving only a small fraction of circulating virus to infect the body in ways peculiar to each virus. To test this possibility, we infused mice intravenously with a harmless common cold virus and tracked its destination by molecular and microscopy methods. Affirming our conjecture, we found that ∼90% of the infused virus homed to the endothelium of the liver and ∼10% went to its macrophages. These data support a unique role, generally underappreciated, for the liver endothelium in viral clearance.

## Introduction

It has been known for more than 50 years that viruses of many sorts when injected into the blood stream of an animal are cleared with astonishing rapidity and efficiency [1]–[6]. Half-lives of decay on the order of 2–4 minutes are the rule; within 10 minutes >90% of circulating virus has been cleared, and within an hour 99%. The phenomenon has been described for nearly all viruses tested; exceptions are rare. The liver, filtering one-third of the cardiac output, is the major organ of initial clearance. The cells implicated are the scavengers of the sinusoids, almost exclusively the Kupffer cell (KC). Cleared virus has been shown to be degraded and non-infectious. The small fraction of virus that escapes clearance is free to infect the host in a manner peculiar to each individual virus.

Of the many viruses studied, one has received special attention. Workers using adenovirus as vectors for gene transfer for genetic vaccine and gene therapy have long known that while these vectors are effectively targeted to chosen tissues, the process is highly inefficient because most of the dose is cleared quickly by the liver. Effective countermeasures to clearance have been elusive, the usual strategies being to increase the dose, to bypass the liver circulation, and to modify the capsid [7], [8]. The basic mechanism underlying rapid and efficient clearance remains unknown. Even the widely held conclusion that the sinusoidal KC is the operant scavenger is not solidly grounded [2]–[5], [9], [10]. In fact, there are many reasons to suspect that the major sinusoidal scavenger is rather the sinusoidal endothelium (liver sinusoidal endothelial cell, LSEC) (see reviews [11], [12]). LSEC are voracious scavengers, more numerous and voluminous than KC; they pinocytose rather than phagocytose, appropriate to taking up virus-sized particles; they contain abundant coated vesicles and lysosomes; and they display receptors for mannose, collagen, hyaluronan, IgG, and scavenger receptors but not complement receptors. An understanding of the basic mechanism underling immediate virus clearance would be of fundamental importance to our knowledge about the hepatic outpost of the immune system, viral pathophysiology and gene transfer vector biology.

As the initial step in studying the viral clearance mechanism, we have undertaken to test whether, as is generally assumed, the KC is the major cell responsible for the rapid and efficient clearance of virus from the blood stream, or, as we suspect, that the long-overlooked LSEC is the major scavenger. Using the well-known mouse model of adenovirus clearance we document, employing two physicochemically different methods of virus detection, that adenovirus is rapidly and efficiently cleared, and with 4-color fluorescence confocal microscopy we show the virus to be localized chiefly to the LSEC and to a much lesser extent to the KC. We confirmed our results with electron microscopy.

## Results

### Clearance kinetics of recombinant human adenovirus serotype 5 (rAd5) from blood

**Figure 1:**
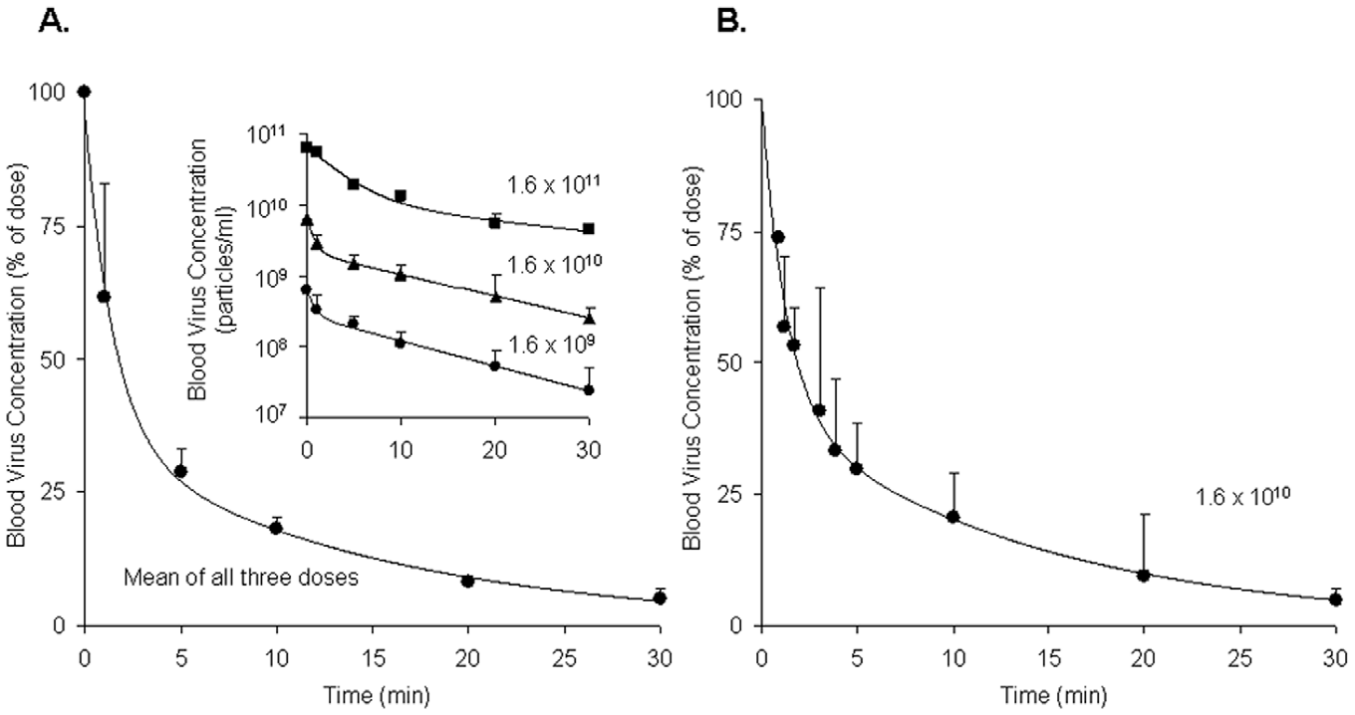
Clearance of rAd5 from mouse blood circulation. We infused by tail vein 3 different doses (1.6×10^11^, 1.6×10^10^ and 1.6×10^9^ DRP) of rAd5, and then measured virus clearance from retroorbital sinus blood by qPCR assays, estimating the viral blood concentration at zero time as the dose divided by the blood volume. **Panel A**: Time course of blood virus concentration. The curve describes the mean percent of dose ± SD cleared over time using data averaged from all 3 administered doses as they were not statistically different. Since the blood concentrations of virus after all three doses decreased in a biphasic fashion, a biexponential decay model was employed to fit the viral concentration-time curves. The insert graph displays the log of blood virus concentration *vs* time for each of the 3 administered doses. Each data point represents mean ± SD of three mice. **Panel B**: Time course of blood virus concentration including “early time points” after the dose of 1.6×10^10^ DRP. Mice of a separate cohort were used to characterize precisely the viral clearance during 0–5 min period. In order to generate a complete concentration-time profile of viral clearance up to 30 min, the concentrations at later time points (10, 20, and 30 min), previously obtained (Panel A insert, 1.6×10^10^), were normalized to the mean concentration at 5 min using the concentration-decay ratio among time points. The complete curve also followed a biexponential decay model. Each data point represents mean ± SD of three mice.

Borrowing a simple mouse model for Ad5 clearance [3], [4], we measured the rate of clearance of virus from mouse blood by infusing intravenously three different doses of replication-incompetent rAd5 and sampling retro-orbital blood periodically over 30 minutes, quantifying viral concentrations by quantitative real-time PCR (qPCR). Estimating the rAd5 blood concentration at zero time as the dose divided by the blood volume, we plotted both the log (Fig. 1A inset) and the mean percent (Fig. 1A full) of virus concentration vs time. We noted a rapid biexponential clearance (*t*_½_ 1 and 10 minutes) of virus from the bloodstream with ∼70% cleared in 5 minutes and >90% in 20 minutes. Sampling blood frequently during the initial 5 minutes after infusion defined the steep first decay phase (Fig. 1B). Clearance of Cy3-labeled rAd5 was more rapid and extensive (see M&M). The decay rate was not different in antibody-deficient mice (Rag2 KO) suggesting that clearance was antibody-independent (not shown).

### Quantification of rAd5 in various organs

Documenting that infused virus homes mostly to liver, we autopsied 3 mice 5 minutes after infusion of 10^11^ rAd5 and quantified organ concentrations by qPCR. Of the total administered dose we recovered 68% in liver, 2% in lung, 1% in spleen, 0% in kidney, leaving 29% unaccounted for, an amount equal to the blood virus concentration at 5 min in Fig. 1. Thus, nearly all cleared virus (97%) was found in the liver.

### Useful antibodies for identifying LSEC and KC in tissue sections

Requiring for our study a precise and reliable method for distinguishing LSEC from KC by immunofluorescence in liver sections, we tested several antibodies to endothelial and macrophage markers (Tables 1 and Table S1). We found two antibodies that identified LSEC reliably. One was mab 2.4G2 anti-FcγRII/III/IV that identifies only FcγRII on these cells based on our observation that LSEC in liver sections from FcγRII-deficient mice showed no fluorescence with this antibody. A second antibody to this marker, anti-LY17.2, was also useful. The other reliable anti-LSEC antibody was rabbit anti-mannose receptor (MR), a result confirmed with a goat antibody to the MR cytoplasmic tail. These anti-LSEC antibodies bound Kupffer cells either very weakly or not at all. The traditional endothelial cell markers tested on LSEC were negative, weak, or inconsistent. It is noteworthy parenthetically that while antibody to the classical endothelial marker, CD31, gave only a very weak signal with LSEC of Balb/C mice in our studies and those of others [13], [14], LSEC from a different strain of mice, C57BL/6, were brightly positive, as others have reported [15] and as has been seen in Sv/129 mice [16].

**Table 1:** Endothelial and macrophage markers employed to distinguish LSEC from KC by immunofluoresence in mouse liver sections.

Antigen^a^	Liver Cells^b^		
	LSEC	KC	V
FcγRII/III/IV^c^ (2.4G2)	+++	+/−	−
MR	+++	+/−	+++
RIIb (Ly17.2)	++	−	−
vWF^d^	+++	−	+++
Cav1	+/−	−	+++
Stabilin 2^e^	+/−	−	−
Endomucin^e^	+/−	−	−
CD31	+/−	−	+
CD34	−	−	−
Flk1	−	−	−
PLVAP	−	−	−
mSIGNR-1	−	−	−
MOMA-1	−	−	−
CD68	−	+++	−
F4/80	−	+++	−
Sialoadhesin	−	+++	−
Mac1	−	+/−	−

a The abbreviations used are: RIIb, Fcγ receptor IIb; MR, mannose receptor; vWF, von Willebrand factor; Cav1, Caveolin 1.

b LSEC were defined morphologically in DIC images as a thin cellular layer lining the sinusoidal lumens. KC were definable by the markers employed and by their situation in sinusoids on LSEC. The veins (V), either portal or central, were identifiable by their large luminal size in DIC images. Immunofluorescence intensity was graded on a subjective +/− scale as follows: +++, intense; ++, moderate; +/−, weak and inconsistent; -, no fluorescence above background. Sections of spleen and yolk sac were used as positive controls for antibodies directed at macrophages and endothelial cells, respectively. Note that some endothelial cell markers label the liver veins.

c Sections of RIIb KO liver show no LSEC fluorescence with mab 2.4G2 anti- FcγRII/III/IV, indicating that only RIIb and not FcγRIII/IV are expressed on LSEC in WT liver; however, KC staining remains +/− in the RIIb-KO liver indicating KC expression of FcγRIII/IV. RIIb expression in KC cannot readily be assessed by this strategy.

d The labeling was not consistent among the triplicate livers with this antibody.

e No consistent labeling within all LSEC and all lobes of liver with this antibody.

For detecting KC we found mabs anti-CD68 and F4/80 to be most reliable. Anti-sialoadhesin as well gave a strong signal with KC and anti-Mac1 a much weaker one (Table 1). Hepatocytes were negative with all of our probes.

### Colocalization studies on LSEC- and KC-distinguishing antibodies

**Figure 2:**
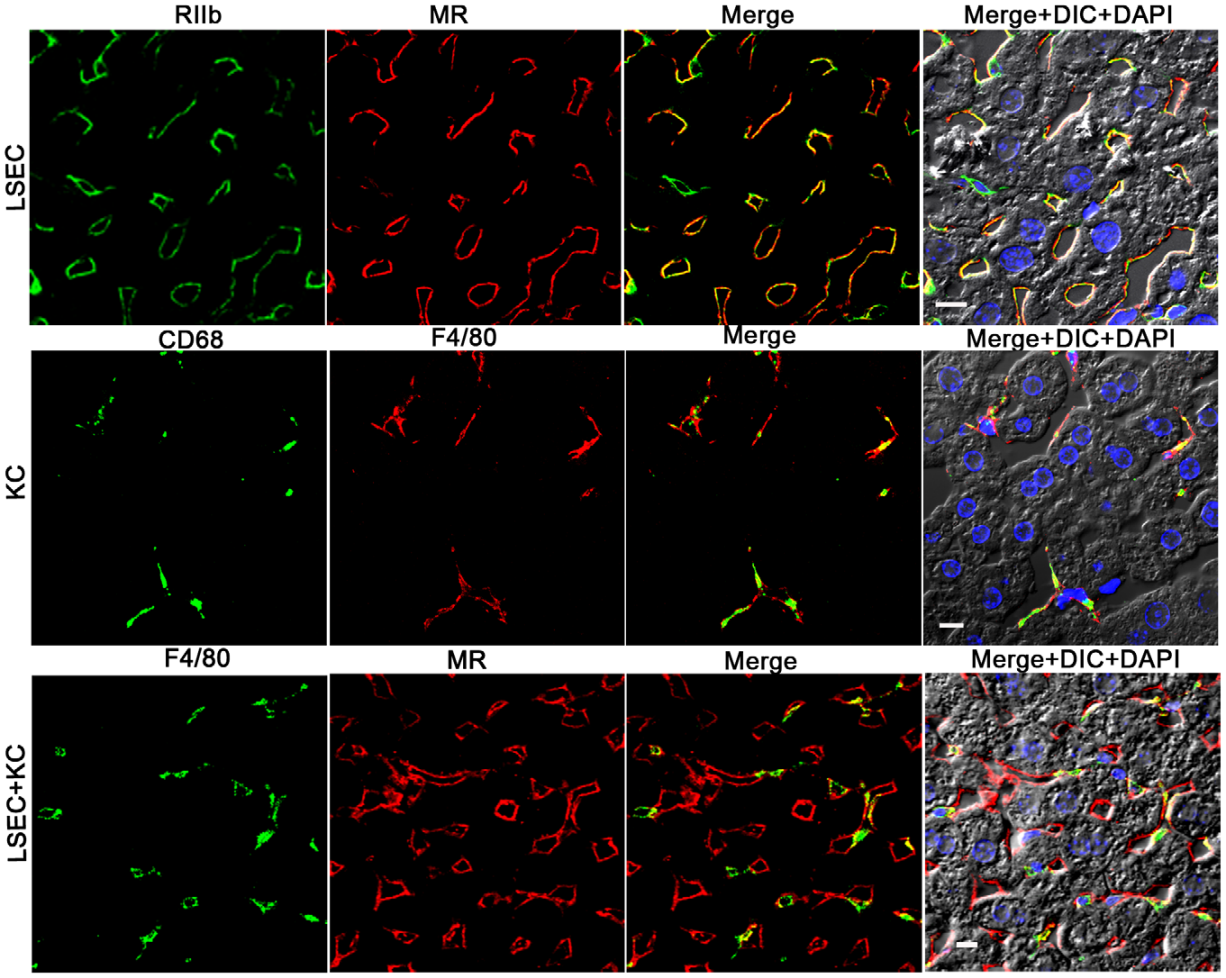
Three-color immunofluorescence images showing LSEC and KC markers in mouse liver. The top row of 4 panels shows LSEC markers, with mab 2.4G2 (green) identifying RIIb in the first column and anti-MR (red) in the second column. The middle row shows KC markers with anti-CD68 (green) in the first column and anti-F4/80 (red) in the second column. The bottom row illustrates the relative proportion of LSEC (labeled with anti MR, first column) and KC (labeled with anti-F4/80, second column). Column 3 shows merged images of the first two columns. Column 4 shows the merged color images plus differential interference contrast image (DIC) and DAPI staining of nuclei (blue). The bars in the panels of column 4 indicate 10 µm. Movies of 3D reconstructions of similar images, illustrating the distribution and abundance of LSEC and KC, are shown in Supplementary Videos S2 and S3, respectively.

Colocalization studies using 2-color confocal immunofluorescence confirmed our choice of antibodies for identifying and distinguishing LSEC and KC. Identifying LSEC simultaneously with mab 2.4G2 (green) and anti-MR (red) gave largely coincidental sinusoidal patterns in green and red and in the merged image, although the cell-associated intensities of the two colors varied independently (Fig. 2, top row of 4 images). Likewise, the 2 KC markers, CD68 (green) and F4/80 (red), reliably identified the same cells as illustrated by near-coincidental patterns in green and red and by the merged image (Fig. 2, middle row of 4 images). We calculated that both colored markers were expressed in 97% of KC. Two-color immunofluorescence with antibodies to LSEC (green) and KC (red) indicated that the two cells can easily be distinguished by this method (Fig. 2, bottom row of 4 panels). Further, it is apparent from this image that the KC are relatively sparse compared to LSEC (see also Supplementary videos S2 and S3), as has been discussed by others [11], [17].

### rAd5 localizes mainly in LSEC

**Figure 3:**
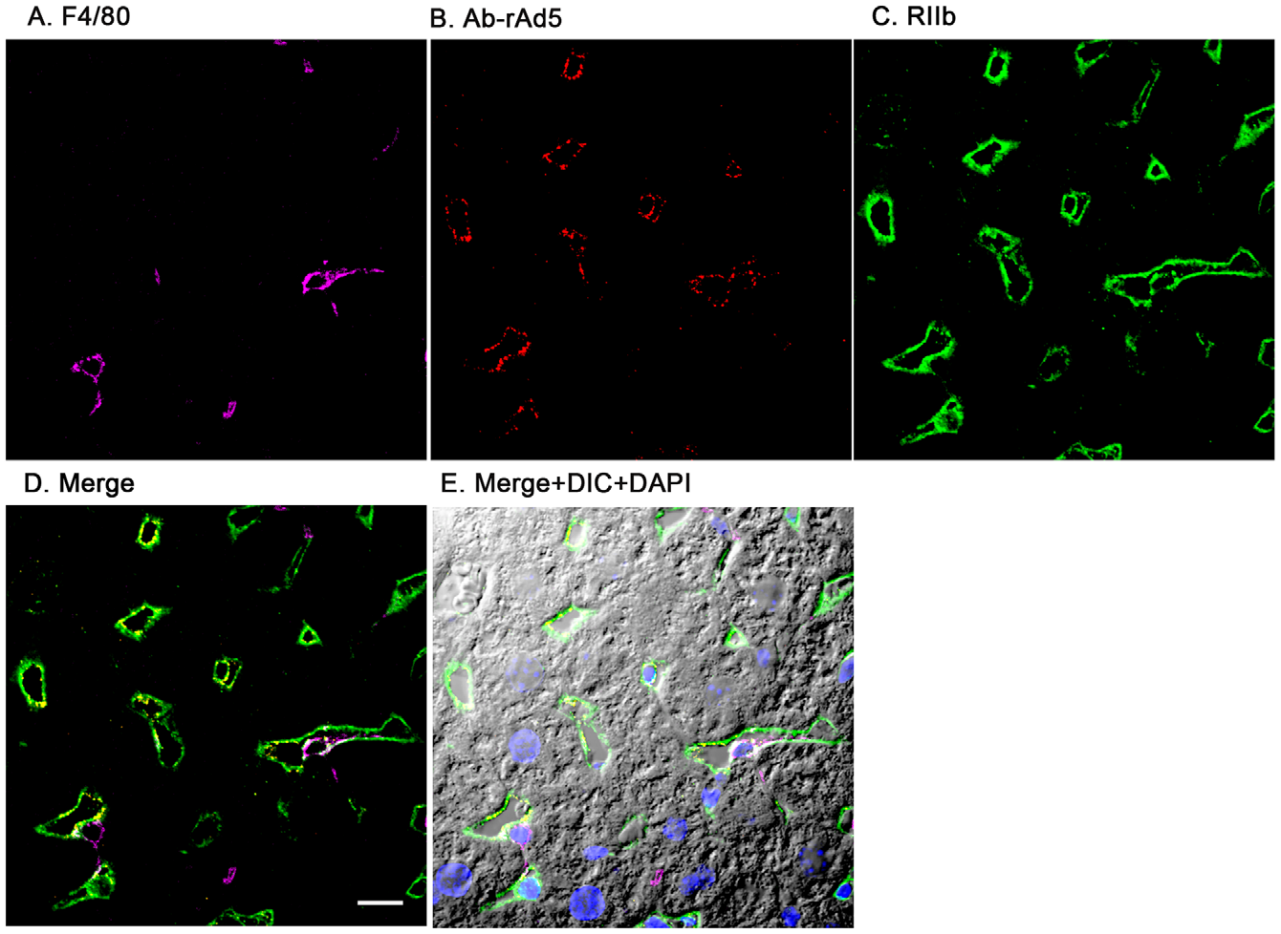
rAd5 labeled with antibody or Cy3 localize predominantly to LSEC. Two sets of 5 images are shown, each set from a separate 4-color fluorescence study in which the identifying markers were varied. Livers from mice infused 1 minute earlier with either 1.6×10^11^ rAd5 particles (left set) or 10^11^ Cy3-labeled rAd5 particles (right set) were fixed, sectioned (5 um), and examined by 4-color fluorescence microscopy using mab anti-F4/80 to label KC; rabbit anti-Ad5 or Cy3 to identify virus, anti-RIIb mab 2.4G2 or anti-MR to identify LSEC; and DAPI to label nuclei. Images were collected with an Olympus FluoView 1000 Laser Scanning Confocal microscope equipped with a spectral detection system (FV 1000 spectra). Representative ∼700 nm optical sections showing typical labeling patterns are presented here. **A**. Magenta color delineates the KC. **B**. Red puncta identify rAd5 particles. **C**. Green mab 2.4G2 and rabbit IgG anti-MR mark LSEC. **D**. Merged images of A, B, and C. **E**. Merged panels A, B, C with DAPI showing cell nuclei (blue) plus DIC defining tissue structure including sinusoidal lumens. The bars in panels E signify 10 µm. Videos of 3D projections from similar images are shown in Supplementary Videos S1–S8.

**Figure 4:**
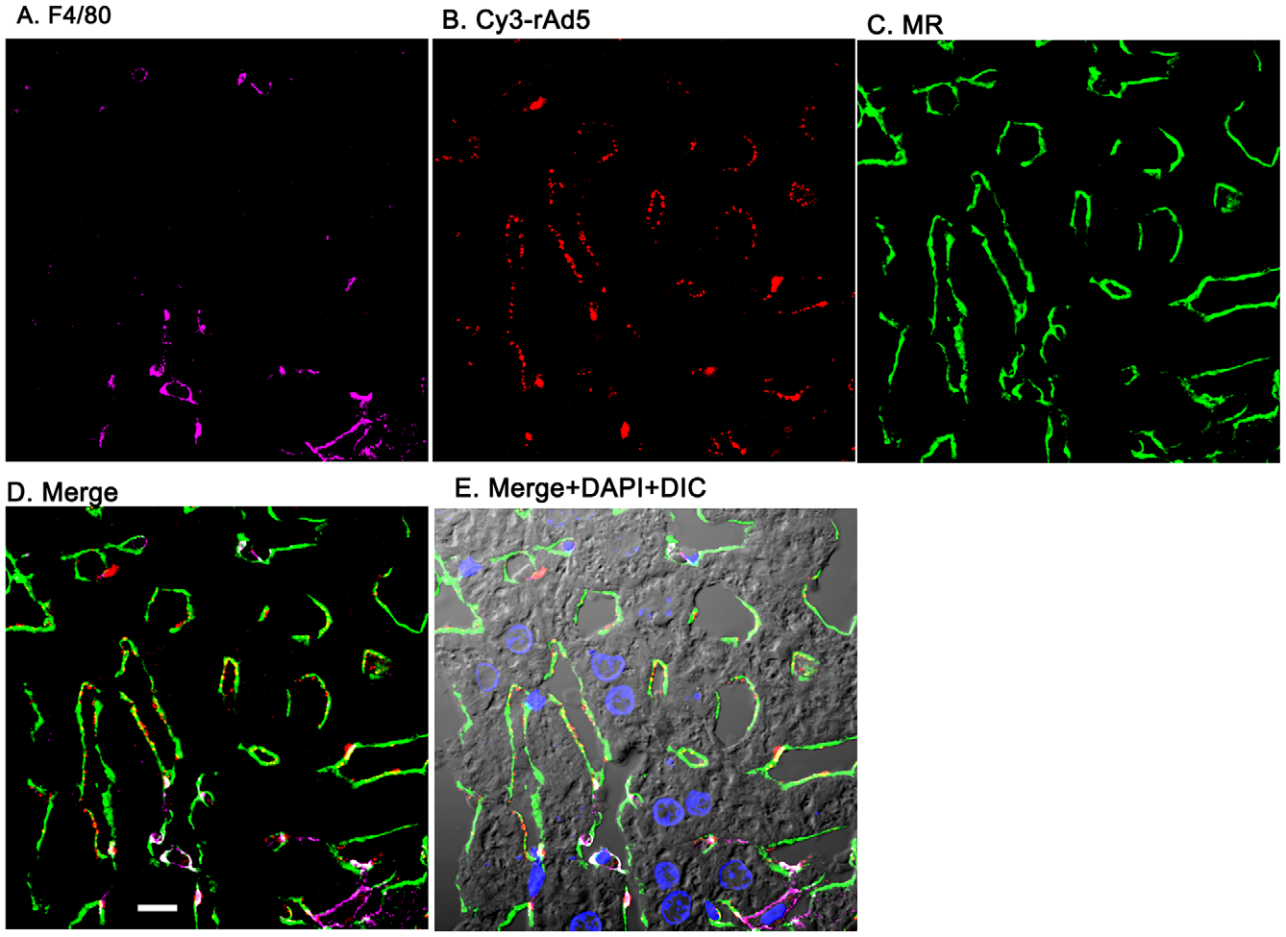
Cy3-labeled rAd5 localize predominantly to LSEC. Livers from mice infused 1 minute earlier with 10^11^ Cy3-rAd5 particles were fixed, sectioned (5 µm), and examined by 4-color fluorescence microscopy using mab anti-F4/80 to label KC; anti-MR to identify LSEC; and DAPI to label nuclei. Cy3 fluorescence identified virus. Images were collected and presented as described in legend to Fig. 3. **A**. Magenta color delineates the KC. **B**. Red puncta identify Cy3-rAd5 particles. **C**. Rabbit IgG anti-MR mark LSEC. **D**. Merged images of A, B, and C. **E**. Merged panels A, B, C with DAPI showing cell nuclei (blue) plus DIC defining tissue structure including sinusoidal lumens. The bars in panels D signify 10 µm. Videos of 3D projections from similar images are shown in Supplementary Videos S1–S8.

Having documented that nearly all blood-borne virus is cleared by the liver, we asked the whereabouts of liver rAd5 at 1 and 10 minutes after infusion, examining fixed liver sections by fluorescence confocal microscopy. rAd5 was detected either by the use of a rabbit anti-Ad5 antibody (red) applied to the liver sections (Fig. 3) or by covalently tagging rAd5 with a Cy3 fluorophor (red) prior to infusion (Fig. 4). LSEC were tagged green with antibodies to FcγRIIb (Fig. 3) and MR (Fig. 4), while KC were marked with anti-F4/80 (magenta). The same experiments were performed identifying KC instead with anti-CD68 (Supplementary Figs. 1&2). In all of these studies, at 1 minute after infusion we detected abundant virus, appearing as red fluorescent puncta with the sinusoids. The vast majority was coincident with LSEC while lesser numbers localized to KC. No virus was associated with hepatocytes, endothelium in large vessels or sinusoidal lumens.

### Quantification substantiates predominant LSEC localization of rAd5

**Figure 5:**
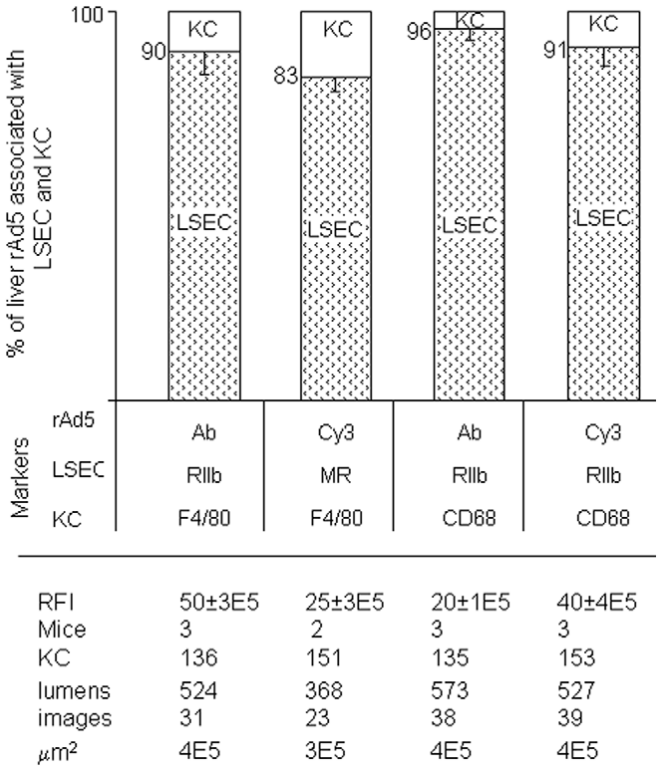
Quantification of rAd5 association with LSEC and KC. We assessed the whereabouts of rAd5 in 4-color fluorescence images like the representative ones shown in Fig 3, Fig 4 and Supplementary Figs S1 and S2, employing 4 combinations of complementary markers to identify cells and virus. By counting total virus color (pixel area x mean intensity) associated with both LSEC and KC, we found that 83–96% of virus was associated with LSEC and 4–17% with KC and none with hepatocytes, as shown in the bar graphs as mean ± SD for each of 4 different experiments. Data are graphed for both strategies for labeling virus, both Cy3-labeling (Cy3) and labeling with anti-Ad5 antibody (Ab). Two different markers identified both LSEC and KC. The table below the Fig illustrates that the 4 experiments were comparable by several parameters including relative fluorescence intensity scored RFI, numbers of mice used (mice), numbers of KC and sinusoid lumens scored (lumens), number of images examined, and the area of tissue examined microscopically (µm^2^).

From images such as those in Fig. 3, Fig. 4 and Supplementary Figs. S1 & S2 we quantified the percent of virus associated with both KC and LSEC (Fig. 5). We found that ∼90% of virus localized to LSEC whereas only ∼10% associated with KC, documenting our visual impressions. The two physicochemically different methods of tagging the virus gave comparable results (all 4 bars of Fig. 5). Animations of 3-color images of liver sections projected in 3-dimensions further document that virus associates predominantly with LSEC (Supplemental videos S1–S8). We incidentally note the subtle suggestion of a difference in appearance of Cy3-labeled (but not antibody-labeled) virus in each of the two cells, LSEC and KC: LSEC-associated virus appears as fine strands of discrete individual particles while in some KC the virus looks patched and clumped.

### Electron microscopy confirms fluorescence result

Confirming by electron microscopy our immunofluorescence results, we examined sections of 39 sinusoidal lumens from livers of 2 mice infused with rAd5 1 min prior to hepatectomy, identifying 34 putative virus particles (round, smooth, dense, ∼100 nm diameter) situated within LSEC but none in KC (total 3 KC identified), whereas 30 sinusoidal lumens from livers of 2 uninfused mice evidenced 2 such particles in LSEC and none in KC (1 KC identified).

## Discussion

We interpret our data to indicate that in the mouse it is the LSEC and not the KC that clears the bulk of blood-borne human adenovirus. This conclusion is based first on our clearance studies documenting that >90% of infused virus has disappeared from the blood within 20 minutes. Second, it is clear from our data and those of others that the liver is responsible for most of the rapid and extensive clearance [1], [2], [4], [5]. Third, of the virus found in the liver 1 minute after infusion, we find ∼90% localized to the LSEC with ∼10% appearing associated with the KC (Fig. 5). Fourth, likely the virus was endocytosed and degraded by the LSEC and KC, as we detect very little Ad5 in the liver at 10 minutes. Others have noted similar rapid disappearance from liver of anti-virus activity [1] and Ad5 genome [4], although it must be acknowledged that some minor portion of cleared virus may escape degradation to replicate or transcytose or be otherwise processed by the scavenging cell.

Thus our data support the contention that the LSEC and not the KC is the major cell type involved in clearing adenovirus from blood during the early minutes after virus infusion. Early 20^th^ century biologists who described the reticuloendothelial system (RES) [18] would have embraced our conclusion, for they appreciated that liver sinusoidal endothelium was vigorously endocytic. However, the predominant view of today favors the KC as the operant liver scavenger of virus [2]–[5], [9], [10]. A possible explanation for this historical change in thinking has been presented by Smedsrod and colleagues (reviewed [12]): During the middle part of the 20^th^ century most biologists dismissed the ‘endothelial’ portion of the RES. Rather, RES function was reconceptualized in terms of a system of mononuclear phagocytes (MPS) [19], the hallmark of which was the capacity to phagocytose. The KC, which pinocytosed but was most characteristically phagocytic, flourished as virtually the sole sinusoidal scavenger. The LSEC, highly pinocytic but incapable of phagocytosis, appeared to lose its consideration as a liver scavenger. Popular reviews and textbooks minimized LSEC scavenging in favor of KC [6], [20], [21]. In the 1980s the Society and Journal of the RES were renamed the Society and Journal of Leukocyte Biology, events that we interpret as manifestations of this cultural shift in view [22].

Only recently has the concept of sinusoidal scavenging been amended: Smedsrod and colleagues, using modern techniques to repeat the vital stain clearance studies of early 20^th^ century [23], [24], demonstrated that LSEC pinocytose more vigorously than KC. These workers have suggested that the old term RES with respect to the liver be thought of functionally as the MPS (the KC) plus the LSEC [12]. Our data showing vigorous adenovirus uptake by LSEC strongly support this renaissance of Aschoff's RES.

Whether serum factors are required for clearance of rAd5, we know only that immunologically naïve animals were used and that clearance rate was unchanged in Rag2-deficient mice, which would indicate that antibodies were not required. Prior workers have not dealt with this issue systematically and those who have approached the question are at odds. One laboratory noted that Newcastle disease virus was cleared ten-times more rapidly when combined with antiserum [2], but another has shown that the half-life of SIV decay with antibody was virtually unchanged [5]. The question of antibody-mediated clearance is especially relevant to our data because receptors for IgG (FcγRII) are abundant on LSEC and are known to take up immune complexes [25], [26].

Although all of the relevant studies on the clearance of blood-borne virus utilized the contrived modality of intravenous infusion (cited above), these data should nevertheless be applicable, one would infer, to the clearance of blood-borne virus during any viremic phase of a natural viral infection. Free virions during viremia should be cleared quickly and effectively by the liver just as when infused unless the clearance mechanism is modified by the adaptive immune system. What fraction of total blood-borne virus during a viremic episode of natural infection remains free of immune system modification has not been systematically studied, insofar as we can discern, and remains a worthy challenge. Thus, it appears currently impossible to estimate directly the magnitude of the ‘free virus’ clearance mechanism during the viremia of natural infection. Nevertheless, for these reasons it would seem desirable to explore the mechanism by which the liver clears blood-borne virus after intravenous infusion. The mechanism may yield new strategies for accelerating clearance for therapeutic purposes. Our data suggest that studies should begin with the LSEC.

Beyond our conclusion that virus is being removed from blood largely by LSEC we can only infer the mechanism of clearance. Note that it is commonly thought that viruses are small enough to be pinocytosed, a process dedicated to small particles (<0.2 µm), and do not require a phagocytic mechanism generally reserved for large particles (>0.5 µm) [27]. The LSEC almost certainly are endocytosing by the process of pinocytosis, as they are well known to pinocytose vigorously, whereas they phagocytose larger particles only under unusual conditions [12]. Further we would suppose that KC, which are capable of both pinocytosis and phagocytosis, would be taking up virus by pinocytosis were the particles monodisperse, but the appearance of some clumped Cy3-virus in KC (Fig. 5) suggests that the labeling process may induce limited aggregation which by size alone would encourage phagocytosis by KC.

Using the strategy described herein for showing LSEC uptake of virus we should be able to test the prediction that other small particles such as small immune complexes, nanoparticles, and others, are taken up in a like manner by the LSEC. Further, now that we have identified the virus-clearing cell, studies investigating the mechanism of clearance and the fate of endocytosed virus should readily follow.

## Methods

### Ethics statement and animals

Wild type male Balb/C mice of age 12–15 weeks were obtained from Taconics Laboratory. This study was carried out in strict accordance with the recommendations in the Guide for the Care and Use of Laboratory Animals of the National Institutes of Health. The protocol was approved by The Ohio State University Institutional Animal Care and Use Committee. All surgery was performed under Isoflurane anesthesia, and all efforts were made to minimize suffering.

### Preparation of recombinant Ad5 (rAd5)

Recombinant adenoviral particles, E1 and E3 deleted Ad5.CMV.eGFP vector (Q-Biogene), were propagated and amplified in HEK 293 A cells (Microbix), cultured in Corning hyper flasks with 10% CCS DMEM. The cells were lysed after 72 hr or once they reached the maximum cytopathic effect by 4x freeze-thaw in the presence of 0.1% Triton X-100 and 25 units/ml Benzonase in TMN 200 buffer (20 mM Tris pH 8, 1 mM MgCl_2_ and 200 mM NaCl). Adenoviral particles from the supernatant were purified from defective viral particles using two CsCl density gradients. First, a discontinuous step gradient was prepared by loading 20 ml of the clarified viral lysate into a 34 ml polyallomer ultracentrifuge tube, underlaying with 6 ml of a 1.2 g/ml CsCl solution, followed by 8 ml of a 1.4 g/ml CsCl solution. Tubes were centrifuged in a SW28 rotor at 23,000 rpm for 90 min at 4°C. The banded virus (∼3 ml) at the 1.2/1.4 g/ml CsCl interface was collected and transferred to a 13 ml ultracentrifuge tube, and approximately 10 ml of 1.35 g/ml CsCl solution added to completely fill the tube. An isopycnic gradient was generated using a NVT65 rotor at 60,000 rpm for 4 hours. The lowest bluish white band containing the purified infectious virus was collected and dialyzed over night against TMN 200 buffer with a minimum of 4 volume exchanges. The rAd5 viral particles were stored in TMN 200 containing 0.1 volume of 50% sucrose at −80°C.

### Titration of rAd5 vector

The DNase resistant viral particle (DRP) rAd5 titer (measuring DNA containing viral particles) was determined by a method previously described for AAV particle titers by our group [28]. Briefly, the rAd5 DRP titers were determined by diluting the viral preparation 100-fold and digesting with DNase 1 (115 units/100 µl) in a Peltier Thermocycler PTC 2000 at 37°C for 30 min followed by 95°C for 10 min. This step removes unencapsidated viral DNA if present. DNase resistant particles were incubated with 10 µg proteinase K incubation at 50°C for 60 min followed by 20 min incubation at 95°C to inactivate Proteinase K. This step degrades the nuclease and viral capsid proteins exposing rAd5 genomic DNA for detection. Digested samples were then subjected to Taqman qPCR by diluting the samples 10 fold (final reaction volume of 25 µl) using Tagman master mix (Applied Biosystems) and the following adenovirus E2A gene primers and probe: E2A forward Primer-5′TTG CGT CGG TGT TGG AGA T 3′ (300 nM); Reverse primer- 5′ CAA GGC CAA GAT CGT GAA GAA; Tagman probe 5′ (6∼FAM) TGC ACC ACA TTT CGG CCC CA (TAMRA-Q) 3′ (150 nM). Concurrently, an adenovirus DNA plasmid standard curve (pHelp adenovirus plasmid containing E2A gene) was set up in triplicate using 10^4^–10^10^ rAd5 genome equivalents. Tagman quantitative PCR assay was performed on the ABI PRISM Sequence Detector 7900HT (PerkinElmer) using Sequence Detector version 1.7 software. Reaction conditions were as follows: 50°C for 2 min, 95°C for 10 min, and 40 cycles of 95°C for 15 s and 60°C for 1 min. Fluorescence emission was plotted as a function of increase in reporter fluorescence (Δ R_n_) versus copy number. The data were analyzed using the default settings of the software for determining baseline and the threshold value. For every assay a standard curve for the primer set was generated and used by extrapolation to estimate the copy number of target sequences in unknown samples. As a general confirmation of the DNA containing DRP vector particle titer, purified rAd5 particles were lysed in lysis buffer (0.1% SDS, 10 mM Tris, pH 7.4, and 1 mM EDTA) and the optical density (OD) at 260 nm measured where an OD of 1 corresponds to 1.1×10^12^ virus particles. As expected, the DRP and OD_260_ titers of Ad5.CMV.eGFP vector were in general agreement (4.1×10^12^ DRP/ml vs 1.4×10^12^ VP/ml, respectively).

Lastly, to confirm that the particles isolated by double CsCl banding were highly infectious, a TCID_50_ infectious unit (IU) endpoint titration assay was performed. The high throughput method consists of detecting replicating rAd5 DNA using E2A qPCR following a 3 day infection of HEK 293 cells. Briefly, 10-fold serial dilutions of a rAd5 stock in replicates of 12 were prepared and used to infect 293 cells in a 96-well plate format. After a 72 hr incubation period, cells were directly lysed with a detergent buffer (1 mM Tris pH 8.0, 1 mM EDTA, 0.5% DOC, 1% Tween 20, 0.1% SDS, 1 mg/ml Proteinase K) and sequentially incubated at 37°C for 1 hr, 55°C for 2 hr, and 95°C for 30 min. The plate was centrifuged briefly to clarify the crude cell lysate (2,000 rpm for 1 minute), and a 1∶4 dilution of the lysate in 1X Taqman PCR Buffer was prepared (this step dilutes out cellular PCR inhibitors). The diluted sample (2.5 ul) was then added to a standard 25 µlE2A qPCR reaction and Taqman E2A qPCR performed to detect the presence of rAd genomic DNA as described above. Genomic rAd5 copies are extrapolated from a plasmid DNA standard curve (pHelp plasmid), and total copies present in the well calculated as follows: total lysate volume per well is 185 µl and 2.5 µl of a 1∶4 lysate dilution was used in each PCR reaction (185/(2.5×0.25)  = 296). Coupled with a Limit of Detection (LOD) of 10 rAd5 genomic copies per qPCR reaction (based on the lowest standard on the curve), a minimum endpoint input value corresponds to 2,960 copies/lysate. Therefore at the assay endpoint of 1 infectious unit in an individual well, greater than 2,960 genomic copies/lysate are required to score the well as positive based on our assay sensitivity. Once scored, the data are then applied to the Karber equation to determine the TCID_50_ of the sample. Significantly, the human Ad5 Reference Material (ARM) [29] was used to qualify the assay; very good agreement between the published ARM IU titer and 96-well qPCR IU titer was obtained (7.0×10^11^ IU/ml ARM vs. 8.2×10^11^ IU/ml E2A qPCR method). Per the ATCC's Product Information Sheet for the Ad5 ARM (Cat. No. VR-1516), the IU titer on HEK 293 cells is 7.0×10^10^ Normalized Infectious Units (NIU)/mL, with 95% certainty that the infectious titer on HEK 293 cells lies within the range of 7.0×10^10^ to 8.0×10^10^ NIU/mL. The average Ad5 ARM TCID_50_ IU titer determined by us using the E2A qPCR method was 8.0×10^10^ IU/ml (assay performed in duplicate). The IU titer of the Ad5.CMV.eGFP was determined to be 1.6×10^12^ IU/ml using the TCID_50_ assay method above, and this yielded a DRP to infectivity ratio of 2.6, indicative of a highly infectious rAd5 preparation. We acknowledge that the calculated IU titer is greater than the calculated OD_260_ titer, which may reflect the inherent variability in all titration methods measuring single particle events, but note that all experiments use the DRP titer to calculate particle inputs.

### Clearance kinetics of infused rAd5 in mice

rAd5 DNase Resistant Particles (DRP) at 3 doses (1.6×10^9^, 1.6×10^10^ and 1.6×10^11^ DRP [3] in 50 µl of PBS pH 7.4 were infused into the tail vein of Balb/C mice, 3 mice for each dose. Mice were bled via the retro orbital plexus of 20 µl blood at time 1, 5, 10, 20 and 30 min. post viral infusions. The zero time was calculated based on the mouse weight and estimated blood volume of 2.58 ml/25 gm (zero time point, particles/ml  =  dose/2.58 ml) [30]. Another set of 3 mice were infused with 1.6×10^10^ DRP and bled *via* the retroorbital plexus at 0.5, 1, 2, 3, 4, and 5 min. Total blood DNA was extracted using DNeasy Blood & Tissue kit (Qiagen). Adenovirus genomes were quantified using qPCR (adenovirus E2A gene as the target) and plotted as the decay of plasma virus vs. time.

### Cy3 labeling of viral particles

CsCl purified particles were labeled according to a modification of the method of Leopold [31]. Briefly, the viral particles at a concentration of 10^12^ DRP/ml in TMN 200 with sucrose were dialyzed overnight with 3 exchanges of PBS pH 7.4. The pH and the molarity of the viral particles in PBS 7.4 buffer was adjusted to 0.05 M sodium carbonate and pH 9.3 by diluting 1∶10 with 0.5 M sodium carbonate pH 9.3. The Cy3 dye was reconstituted with the viral particle solution and incubated for 30 min at room temperature. The reaction was stopped using 0.2% glycine and dialyzed against two changes of TMN 200 at 4°C over 16 hr. Adenoviral particles were stored in TMN 200 containing 1∶10 volume of 50% sucrose at −80°C until use. Cy3 dye concentration was determined by recording the absorbance at 552 nm and using the extinction coefficient provided by the manufacturer. Dye-to-capsomere ratios were calculated on the basis of the surface exposure of 252 viral capsomeres per virion and found to be 0.56 [32]. The viral titer of the fluorophore-conjugated rAd5 was determined as described above using qPCR. The yield of the fluorophore-conjugated Ad viral stock (2.3×10^12^ DRP) was 82% of the parent stock (2.8×10^12^ DRP). Clearance of Cy3 labeled rAd5 was tested in the same manner as the clearance of unlabelled rAd5 (Fig. 1). When time zero viral concentration was estimated as in Fig. 1, 99% of Cy3-rAd5 was cleared at 10 minutes. If time zero was ignored and only blood samples were considered, then between 1 and 10 min 89% of virus was cleared.

### Quantification of rAd5 in various organs

Mice (n = 3) were infused intravenously with 10^11^ viral particles and at 5 min were sacrificed; organs (liver, kidney, lung and spleen) were removed and weighed. DNA was extracted from weighed portions of each organ using DNeasy Blood & Tissue kit (Qiagen) and quantified for rAd5 by E2A qPCR as described earlier.

### Immunofluorescence

The livers were removed as rapidly as possible and small pieces (∼5 mm) were cut and transferred to 4% paraformaldehyde-PBS and fixed for 2 hrs at room temperature. Tissue was washed several times in PBS and then infused in 20% sucrose-PBS overnight at 4°C. The tissue was then embedded in a freezing medium for cryostat sectioning and stored at −80°C until used. Cryostat sections, 5 m thickness, were collected on Superfrost microscope slides (Fisher Scientific, Pittsburgh, PA). The sections were blocked in 5% milk-PBS prior to incubation with primary antibodies overnight at 4°C. The details of all the primary antibodies are given in Table S1. Antibody binding was localized using fluorophore conjugated secondary antibodies in blocking buffer for 1 hr at 4°C. Secondary antibodies used for this studies, namely, goat IgG anti-rat IgG (conjugated with dyes Alexa 647 or 488), goat IgG anti-rabbit IgG (conjugated with Alexa dyes 647, 488 or 594) and Alexa 488-conjugated goat IgG anti-chicken IgY, Alexa 594 dye-conjugated Streptavidin (594-Streptavidin) and Alexa 488 dye-conjugated goat IgG anti-mouse IgG, were purchased from Invitrogen. Dylight 594 conjugated goat IgG anti-rat IgG and FITC conjugated goat IgG anti-Armenian Hamster IgG were bought from Jackson Immuno research. Purified rat IgG was purchased from Santa Cruz Biotechnology. Unless specified all secondary antibodies were used at a 1∶200 dilution. Nuclei were stained with DAPI for 10 min and the sections were mounted under cover slips in Prolong gold (Invitrogen). Control incubations included isotype controls along with their respective secondary antibodies and also secondary antibodies alone.

The above protocol was modified as follows for 4 color imaging and to accommodate two rat antibodies. Livers were removed at 1 minute from mice infused with 1.6×10^11^ rAd5 viral particles and processed as above. 5 µm liver sections were incubated with the rat mab 2.4G2 and the rabbit polyclonal anti-whole Ad5 antibody (Access Biomedicals) [33] at a concentration of 1∶25 in blocking solution, over night at 4°C. Following washing with PBS azide for 1 hr sections were labeled with Alexa 647-conjugated goat anti-rat IgG and Alexa 594-conjugated goat anti-rabbit IgG (Invitrogen). The unbound anti-rat epitopes were masked by incubating with excess normal rat IgG at a concentration of 20 µg/ml. Sections were immediately labeled with the Alexa 488-conjugated rat mab anti-F4/80 or CD68 by incubating over night at 4°C. Subsequently sections were washed for 1 hr using PBS azide and stained with DAPI for 10 min before mounting in Prolong gold. The rabbit polyclonal anti-Ad5 antibody (Access Biomedicals) was tested for background binding in un-infused mouse liver and found to be less than 1% in LSEC and KC. Two other antibodies tested for this study, namely rabbit polyclonal against purified adenovirus type 5 (Abcam) and goat polyclonal anti-adenovirus antibody (AbD Serotech), were found to be unreliable based on their non- specific binding patterns. Liver from mice infused with Cy3 labeled rAd5 were processed in the same manner as above omitting anti-Ad5 antibody and its secondary antibody. To determine CD68 expression of F4/80 positive cells, sections were first labeled with anti-F4/80 followed by Alexa 594 dye-conjugated goat IgG anti-rat IgG, then an excess of rat IgG (10 µg/ml) to block unbound anti-rat epitopes. Subsequently the sections were incubated with Alexa 488 conjugated CD68 and visualized for labeling with two rat antibodies. The sections were examined and images were acquired in the Olympus Fluo view 1000 Laser Scanning Confocal microscope equipped with a spectral detection system for a finer separation of fluorochromes (FV 1000 spectra). Image analysis and 3-dimension z stack reconstructions were done in Fluoview software (version 2.1.39).

### Quantification of virus within liver sections

We determined by inspection of 4-color confocal images that rAd5 was associated only with LSEC and KC, and not with hepatocytes or endothelium in larger vessels. To quantify the relative proportion of virus associated with KC and LSEC we employed Image J software developed in JAVA, using a 3-step procedure. 1. After thresholding the entire image to separate viral pixels from background pixels, we determined the total fluorescence intensity of virus in the entire image. 2. Each KC, identified using anti-CD68 or anti-F4/80 in a microscopic image, was manually segmented by liberally outlining the margins; thresholding was applied and the fluorescence intensity of rAd5 associated with each KC was recorded; the data were summed to obtain the total intensity of KC-associated virus of an image. 3. The total intensity of rAd5 associated with KC was subtracted from the total intensity of rAd5 of the entire image to give the total intensity of rAd5 in LSEC alone. The values for all images were summed. The number of KC analyzed in Fig 3 and Supplementary Fig 2 and 3 are comparable with a ratio of KC per LSEC lumen being 3 to 4 KC. The relative fluorescence intensity (RFI) of the images analyzed with anti-Ad5 antibody is relatively less than the images from Cy3 labeled virus indicating that Cy3 are much brighter than the virus labeled with anti-Ad5 antibody.

### Creation of 3D video animations showing virus association with LSEC and KC

To illustrate in 3-dimensions the association of virus with each of the two relevant cells (LSEC and KC), 7 videos were created using Olympus Fluoview Software (version 2.1.39) from a single 3-color fluorescence image of a liver section prepared as described in the legend to Fig 4. The videos represent z-stacks collected with an Olympus FluoView 1000 Laser Scanning Confocal microscope equipped with a spectral detection system (FV 1000 spectra) and a 60x PLAPON60XOSC lens, which provides for under 0.1–0.2 µm of chromatic aberration when working with lasers from 405 nm up to 650 nm. The z-dimension (9.6 µm) is derived from a stack of 33 optical sections acquired in 0.3 µm increments. The dimensions of the reconstructed stack in x, y and z are 211.5, 211.5 and 9.6 µm, respectively. Videos S1–S3 show virus and the two cells individually. Videos S4–S6 show virus with each of the two cells. Videos S7 and S8 (a zoom of 7) show all three elements together. KC identified by anti-F4/80 appear in pseudo-magenta, although in one video (S6) KC were colored green to enhance the contrast with red virus. LSEC marked with anti-MR are pseudo-green. Virus was labeled with Cy3 and appears red. The movies show a cyclical tilting of about 70°C. Note that the sinusoids near the axis of rotation show greatest definition and detail. A quantification of the virus-cell association derived from images like this is presented in Fig 5.

### Transmission electron microscopy

Electron microscopy sample preparation was based on our previous method with some modifications [34]. Briefly, the livers from mice infused with 1.6×10^11^ viral particles or un-infused were removed after 1 min and cut into 1–2 mm^3^ pieces and fixed in 2% glutaraldehyde in 100 mM cacodylate buffer (pH 7.2) containing 5% sucrose for 1 hr at 22°C and subsequently washed with cacodylate buffer. The tissue was subsequently post- fixed in 2% OsO_4_ in 100 mM cacodylate buffer pH 7.2 followed by dehydydration in graded series of ethanol and incubation with propylene oxide prior to infiltration and embedding in Epon 812 resin. Sections of ∼70 nm were cut using a Leica EM UC6 Ultramicrotome, mounted on grids and contrast-stained with lead citrate and uranyl acetate. Thin sections were then examined using a FEI Tecnai G2 Spirit Transmission Electron Microscope. LSEC were identified as very thin gap-disrupted cells, thick at the nuclei, defining the sinusoidal lumens, overlying the space of Disse. KC were identified as cells in the sinusoidal lumens closely adhering to the LSEC surface.

## Supporting Information

Figure S1 **Ab-labeled rAd5 localizes predominantly to LSEC-using CD68 as marker for KC and RIIb for LSEC.** Liver from a mouse infused 1 min earlier with 1.6×10^11^ DRP rAd5 and analyzed by 4-color fluorescence using mab anti-CD68, rabbit anti-Ad5, mab 2.4G2, and DAPI. **A**. Magenta color delineates KC. **B**. Red puncta identify rAd5 particles. **C**. Green 2.4G2 marks LSEC. **D**. Merge of A, B, and C shows virus confined to LSEC and KC. **E**. Panels A, B, C and DAPI showing cell nuclei plus DIC defining tissue structure including sinusoidal lumens. The bar in panel C signifies 10μm. Quantification of this experiment is included in Fig. 5.
(PDF)

Figure S2 **Cy3-labeled rAd5 confined predominately to LSEC shown using CD68 as marker for KC and RIIb for LSEC.** Liver from a mouse infused 1 min earlier with 10^11^ Cy3-labeled rAd5 viral particles (10^11^ DNAase-resistant particles) analyzed by 4-color fluorescence microscopy using mab CD68 to label KC, mab 2.4G2 to identify LSEC, and DAPI. **A**. Magenta color delineates the KC. **B**. Red puncta of Cy3 identify rAd5 particles. **C**. Green mab 2.4G2 marks the LSEC. **D**. Merge of A, B and C. **E**. Merged panels A, B, C with DAPI showing cell nuclei (blue) plus DIC defining tissue structure including sinusoidal lumens. The bar in panel D signifies 10 μm. Quantification of this experiment is included in Fig. 5.
(PDF)

Table S1 **Details of all antibodies used in this study (complementary to** **Table 1** **).**
(DOC)

Video S1 **Virus (red) alone.** (Quick time; 1 MB) Viruses appear as red puncta often coalescing into a tubular pattern that manifests some directionality, all suggestive of the walls of LSEC-lined sinusoids. Details of image acquisition and 3D video creation are given in Materials and Methods.
(MOV)

Video S2 **LSEC (pseudo-green) alone.** (Quick time; 2.7 MB) Sinusoids cut transversely and longitudinally are lined with thin green LSEC encircling empty sinusoidal lumens. Uncolored hepatocytes fill the spaces between sinusoids. Details of image acquisition and 3D video creation are given in Materials and Methods.
(MOV)

Video S3 **KC (pseudo-magenta) alone.** (Quick time; 407 KB) Several KC are seen, most with long projections. KC are more sparse than LSEC. Details of image acquisition and 3D video creation are given in Materials and Methods.
(MOV)

Video S4 **Virus (red) + LSEC (pseudo-green).** (Quick time; 3 MB MB) The great majority of red virus is either in, on, or very near the green LSEC image suggesting close association of the two. Details of image acquisition and 3D video creation are given in Materials and Methods.
(MOV)

Video S5 **Virus(red) + KC (pseudo-magenta).** (Quick time; 1.3 MB) A minority of virus puncta appear associated with KC. To enhance contrast, we have colored KC green in the next video (S6). Details of image acquisition and 3D video creation are given in Materials and Methods.
(MOV)

Video S6 **Virus (red) + KC (duplicate of video 5 using green for contrast with red virus).** (Quick time; 1.3 MB) In this image alone KC are colored green to heighten the contrast with red virus. It shows that only a minority of virus are associating with KC, some of which are merged yellow. Most viruses are distant from KC. Details of image acquisition and 3D video creation are given in Materials and Methods.
(MOV)

Video S7 **Virus (red) + LSEC (green) + KC (pseudo-magenta).** (Quick time; 3.1 MB) This moving 3D image of all three colored elements confirms that most of the virus associates with the LSEC and a minority with KC. The association of virus with each of the two cell types is better discernable in the 2-color videos (S4–S6) above. It is also apparent that the LSEC is the predominant cell type of the two. The section of a sinusoid marked by a white arrow is enlarged in Video S8. Details of image acquisition and 3D video creation are given in Materials and Methods.
(MOV)

Video S8 **Zoom of a single sinusoidal lumen from Video S7.** (Quick time; 2.6 MB) Note here a section of a hepatic sinusoid defined by walls of green LSEC, densely speckled with red virus, some of which appear yellow having merged with green. The dimensions of the reconstructed stack in x, y and z are 34.5, 28.5 and 9.6 µm, respectively. Details of image acquisition and 3D video creation are given in Materials and Methods.
(MOV)
