# Supplementary figures and images for: Rapid and Efficient Clearance of Blood-borne Virus by Liver Sinusoidal Endothelium
Source: PLoS Pathog. 2011 Sep 29;7(9):e1002281. doi: 10.1371/journal.ppat.1002281 (PMC3182912; doi:10.1371/journal.ppat.1002281)

**A. CD68**

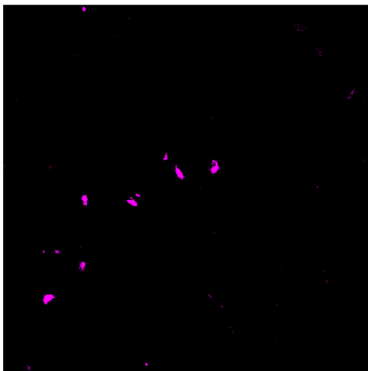

**B. Ab rAd5**

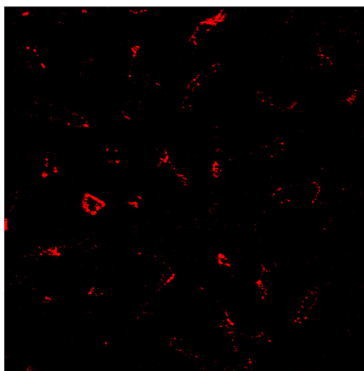

**C. RIIb**

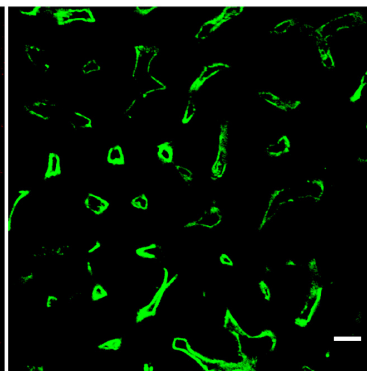

**D. Merge**

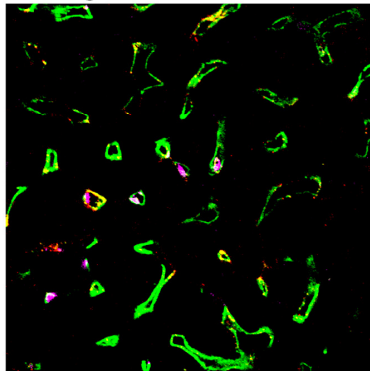

**E. Merge+DIC+DAPI**

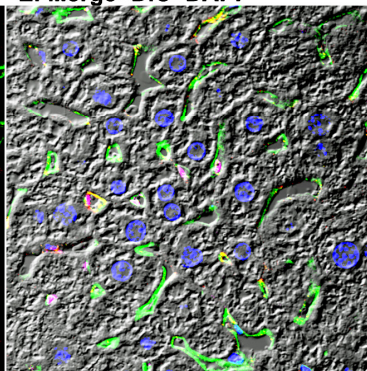

Supplement: Figure S1 — Ab-labeled rAd5 localizes predominantly to LSEC-using CD68 as marker for KC and RIIb for LSEC. Liver from a mouse infused 1 min earlier with 1.6×10^11^ DRP rAd5 and analyzed by 4-color fluorescence using mab anti-CD68, rabbit anti-Ad5, mab 2.4G2, and DAPI. A. Magenta color delineates KC. B. Red puncta identify rAd5 particles. C. Green 2.4G2 marks LSEC. D. Merge of A, B, and C shows virus confined to LSEC and KC. E. Panels A, B, C and DAPI showing cell nuclei plus DIC defining tissue structure including sinusoidal lumens. The bar in panel C signifies 10μm. Quantification of this experiment is included in Fig. 5. (PDF) [file ppat.1002281.s001.pdf]

**A. CD68**

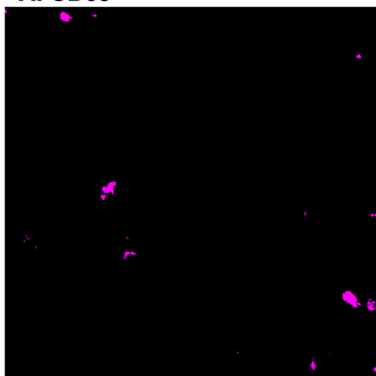

**B. Cy3 rAd5**

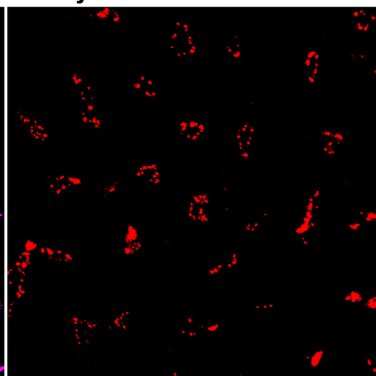

**C. RIIb**

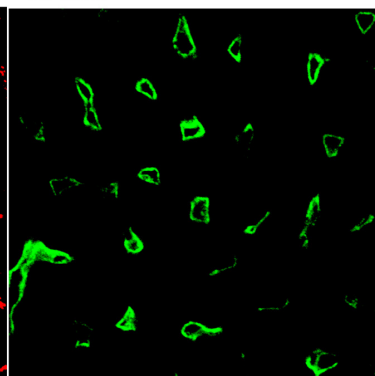

**D. Merge**

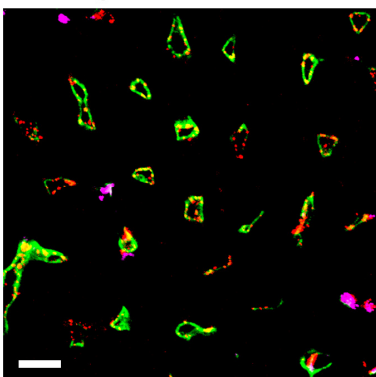

**E. Merge+DIC+DAPI**

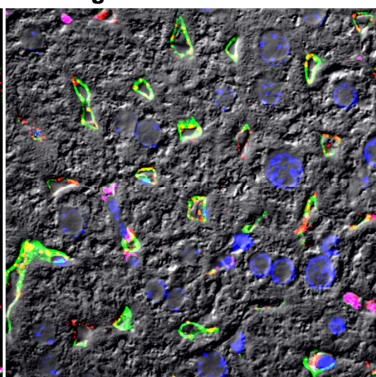

Supplement: Figure S2 — Cy3-labeled rAd5 confined predominately to LSEC shown using CD68 as marker for KC and RIIb for LSEC. Liver from a mouse infused 1 min earlier with 1011 Cy3-labeled rAd5 viral particles (1011 DNAase-resistant particles) analyzed by 4-color fluorescence microscopy using mab CD68 to label KC, mab 2.4G2 to identify LSEC, and DAPI. A. Magenta color delineates the KC. B. Red puncta of Cy3 identify rAd5 particles. C. Green mab 2.4G2 marks the LSEC. D. Merge of A, B and C. E. Merged panels A, B, C with DAPI showing cell nuclei (blue) plus DIC defining tissue structure including sinusoidal lumens. The bar in panel D signifies 10 μm. Quantification of this experiment is included in Fig. 5. (PDF) [file ppat.1002281.s002.pdf]
