# Supplementary material for: Rapid and Efficient Clearance of Blood-borne Virus by Liver Sinusoidal Endothelium
Source: PLoS Pathog. 2011 Sep 29;7(9):e1002281. doi: 10.1371/journal.ppat.1002281 (PMC3182912; doi:10.1371/journal.ppat.1002281)
Supplement: Table S1 — Details of all antibodies used in this study (complementary to Table 1 ). (DOC) [file ppat.1002281.s003.doc]

**Table S1. Details of all antibodies used in this study (complementary to Table 1)**

| Antigen^a^ | Host^b^ | Clone^c^ | Isotype^d^ | Source^e^ | References |  |
| --- | --- | --- | --- | --- | --- | --- |
| FcγRIIb/III/IV | rat mab | 2.4G2 | IgG2bk | BD Pharmingen | [1] | |
| MR (CD206) | rab, gab | NA | NA | Santa Cruz | NA | |
| LY17 (RIIb) | mouse mab | K9.361.cl5 | NA | Cederlab | [2] | |
| vWF | rab | NA | NA | Abcam | [3] | |
| Cav1 | cab | NA | IgY | Robinson | [4] | |
| Cav1 | rab | NA | IgG | BD Pharmingen | [5] | |
| Stabilin 2 | rab | NA | NA | Peter McCourt | [6] | |
| Endomucin | rat mab | V.7C7, V1A7 | IgG1 | Santa Cruz | [7] | |
| CD31 (PECAM-1) | hamster mab | 2H8 | IgG | BD Pharmingen | [8] | |
| CD34 | rat mab | MEC 14.7 | IgG2ak | Abcam | [9] | |
| Flk1 | rat mab | AVAS 12a1 | IgG2ak | BD Pharmingen | [10] | |
| PLVAP | rat mab | MECA 32 | IgG2ak | Abcam | [11] | |
| mSIGNR-1 | rat mab | ER-TR9 | IgM | AbD serotech, Georg Kraal | [12] | |
| CD209b (MOMA1) | hamster mab | 22D1 | IgG | e-bioscience | [13] | |
| CD68 | rat mab | FA-11 | IgG2a | AbD serotec | [14] | |
| F4/80 | rat mab | C1:A3-1 | IgG2b | AbD serotec | [15] | |
| Sialoadhesin | rat mab | 3D6.112 | IgG2a | Abcam | [16] | |
| Mac1 | rat mab | M1/70.15 | IgG2b | AbD Serotec | [17] | |
| mouse IgG | gab | polyclonal | IgG | Invitrogen | NA | |
| CD146 | rat mab | ME-9F1 | IgG2a | Miltenyi Biotec | [18] | |
| whole Ad5 | rab | NA | NA | Access Biomedicals (CA) | [19] | |

^a^ Molecule or epitope recognized by antibody.

^b^ The host species in which the antibodies were made. Abbreviations: mab, monoclonal antibody; rab, rabbit polyclonal antibody; gab, goat polyclonal antibody; cab, chicken polyclonal antibody.

^c^ The clone name of the antibody. NA, not available.

^d^ The isotype of the antibody or NA, not applicable, if the antibody is polyclonal.

^e^ Source of antibody. Robinson, co-author John M. Robinson; Peter McCourt, University of Tromso, Norway; Dr. Georg Kraal, Vrije University Medical Center, Amsterdam, The Netherlands.

Reference List

1. Unkeless JC (1979) Characterization of a monoclonal antibody directed against mouse macrophage and lymphocyte Fc receptors. J.Exp.Med. 150:580-596

2. Holmes KL, Palfree RG, Hammerling U, Morse HCI (1985) Alleles of the Ly-17 alloantigen define polymorphisms of the murine IgG Fc receptor. Proc.Natl.Acad.Sci. 82:7706-7710

3. Denis C, Methia N, Frenette PS, Rayburn H, Ullman-Cullere M, Hynes RO, Wagner DD (1998) A mouse model of severe von Willebrand disease: defects in hemostasis and thrombosis. Proc.Natl.Acad.Sci.U.S.A 95:9524-9529

4. Lyden TW, Anderson CL, Robinson JM (2002) The endothelium but not the syncytiotrophoblast of human placenta expresses caveolae. Placenta 23:640-652

5. Bush WS, Ihrke G, Robinson JM, Kenworthy AK (2006) Antibody-specific detection of caveolin-1 in subapical compartments of MDCK cells. Histochem.Cell Biol. 126:27-34

6. Hansen B, Longati P, Elvevold K, Nedredal GI, Schledzewski K, Olsen R, Falkowski M, Kzhyshkowska J, Carlsson F, Johansson S, Smedsrod B, Goerdt S, Johansson S, McCourt P (2005) Stabilin-1 and stabilin-2 are both directed into the early endocytic pathway in hepatic sinusoidal endothelium via interactions with clathrin/AP-2, independent of ligand binding. Exp.Cell Res. 303:160-173

7. Cyster JG, Shotton DM, Williams AF (1991) The dimensions of the T lymphocyte glycoprotein leukosialin and identification of linear protein epitopes that can be modified by glycosylation. EMBO J. 10:893-902

8. Graesser D, Solowiej A, Bruckner M, Osterweil E, Juedes A, Davis S, Ruddle NH, Engelhardt B, Madri JA (2002) Altered vascular permeability and early onset of experimental autoimmune encephalomyelitis in PECAM-1-deficient mice. J.Clin.Invest 109:383-392

9. Suzuki A, Andrew DP, Gonzalo JA, Fukumoto M, Spellberg J, Hashiyama M, Takimoto H, Gerwin N, Webb I, Molineux G, Amakawa R, Tada Y, Wakeham A, Brown J, McNiece I, Ley K, Butcher EC, Suda T, Gutierrez-Ramos JC, Mak TW (1996) CD34-deficient mice have reduced eosinophil accumulation after allergen exposure and show a novel crossreactive 90-kD protein. Blood 87:3550-3562

10. Shalaby F, Rossant J, Yamaguchi TP, Gertsenstein M, Wu XF, Breitman ML, Schuh AC (1995) Failure of blood-island formation and vasculogenesis in Flk-1-deficient mice. Nature 376:62-66

11. Pelletier RP, Morgan CJ, Sedmak DD, Miyake K, Kincade PW, Ferguson RM, Orosz CG (1993) Analysis of inflammatory endothelial changes, including VCAM-1 expression, in murine cardiac grafts. Transplantation 55:315-320

12. Engering A, van Vliet SJ, Geijtenbeek TB, van Kooyk Y (2002) Subset of DC-SIGN(+) dendritic cells in human blood transmits HIV-1 to T lymphocytes. Blood 100:1780-1786

13. Kang YS, Kim JY, Bruening SA, Pack M, Charalambous A, Pritsker A, Moran TM, Loeffler JM, Steinman RM, Park CG (2004) The C-type lectin SIGN-R1 mediates uptake of the capsular polysaccharide of Streptococcus pneumoniae in the marginal zone of mouse spleen. Proc.Natl.Acad.Sci.U.S.A 101:215-220

14. Holness CL, da Silva RP, Fawcett J, Gordon S, Simmons DL (1993) Macrosialin, a mouse macrophage-restricted glycoprotein, is a member of the lamp/lgp family. J Biol Chem. 268:9661-9666

15. Morris L, Graham CF, Gordon S (1991) Macrophages in haemopoietic and other tissues of the developing mouse detected by the monoclonal antibody F4/80. Development 112:517-526

16. Oetke C, Kraal G, Crocker PR (2006) The antigen recognized by MOMA-I is sialoadhesin. Immunol.Lett. 106:96-98

17. Springer T, Galfre G, Secher DS, Milstein C (1979) Mac-1: a macrophage differentiation antigen identified by monoclonal antibody. Eur.J.Immunol. 9:301-306

18. Harder R, Uhlig H, Kashan A, Schutt B, Duijvestijn A, Butcher EC, Thiele HG, Hamann A (1991) Dissection of murine lymphocyte-endothelial cell interaction mechanisms by SV-40-transformed mouse endothelial cell lines: novel mechanisms mediating basal binding, and alpha 4-integrin-dependent cytokine-induced adhesion. Exp.Cell Res. 197:259-267

19. Xie J, Chiang L, Contreras J, Wu K, Garner JA, Medina-Kauwe L, Hamm-Alvarez SF (2006) Novel fiber-dependent entry mechanism for adenovirus serotype 5 in lacrimal acini. J.Virol. 80:11833-11851
